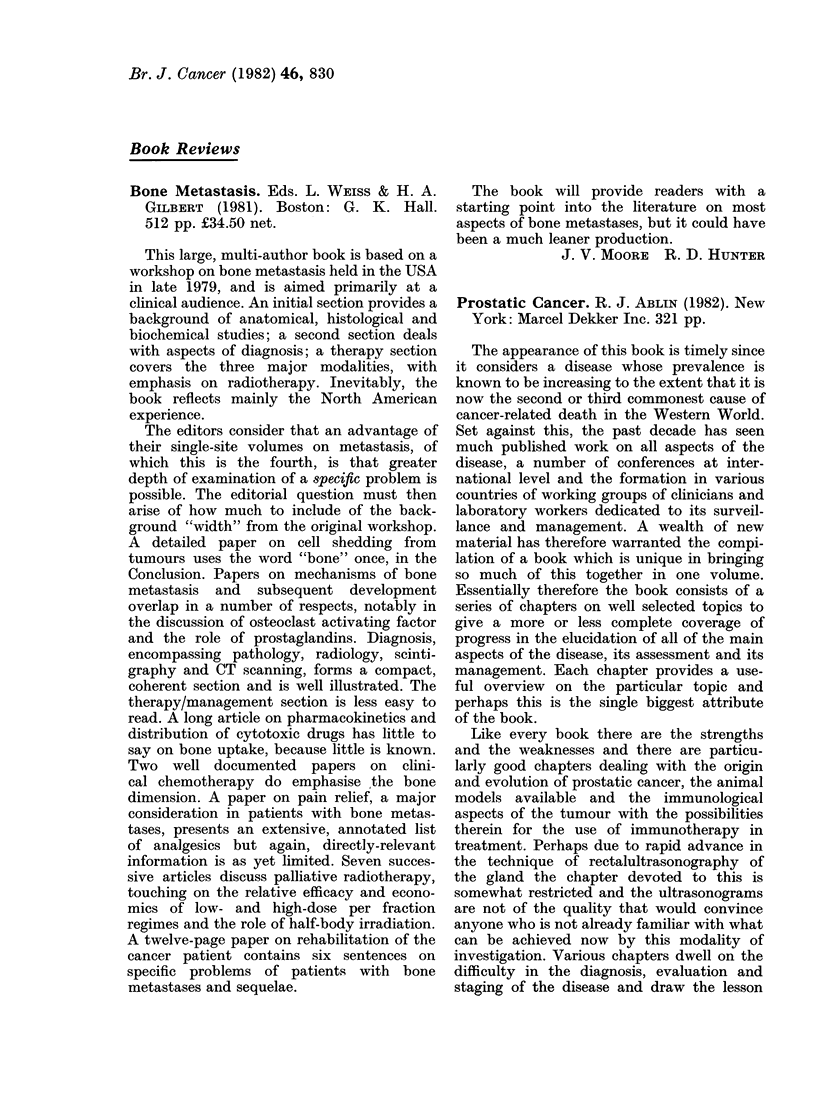# Bone Metastasis

**Published:** 1982-11

**Authors:** J. V. Moore, R. D. Hunter


### *Book Reviews*

**Bone Metastasis.** Eds. L. Weiss & H. A. Gilbert (1981). Boston: G. K. Hall. 512 pp. £34.50 net.

This large, multi-author book is based on a workshop on bone metastasis held in the USA in late 1979, and is aimed primarily at a clinical audience. An initial section provides a background of anatomical, histological and biochemical studies; a second section deals with aspects of diagnosis; a therapy section covers the three major modalities, with emphasis on radiotherapy. Inevitably, the book reflects mainly the North American experience.

The editors consider that an advantage of their single-site volumes on metastasis, of which this is the fourth, is that greater depth of examination of a *specific* problem is possible. The editorial question must then arise of how much to include of the background "width" from the original workshop. A detailed paper on cell shedding from tumours uses the word "bone" once, in the Conclusion. Papers on mechanisms of bone metastasis and subsequent development overlap in a number of respects, notably in the discussion of osteoclast activating factor and the role of prostaglandins. Diagnosis, encompassing pathology, radiology, scintigraphy and CT scanning, forms a compact, coherent section and is well illustrated. The therapy/management section is less easy to read. A long article on pharmacokinetics and distribution of cytotoxic drugs has little to say on bone uptake, because little is known. Two well documented papers on clinical chemotherapy do emphasise the bone dimension. A paper on pain relief, a major consideration in patients with bone metastases, presents an extensive, annotated list of analgesics but again, directly-relevant information is as yet limited. Seven successive articles discuss palliative radiotherapy, touching on the relative efficacy and economics of low- and high-dose per fraction regimes and the role of half-body irradiation. A twelve-page paper on rehabilitation of the cancer patient contains six sentences on specific problems of patients with bone metastases and sequelae.

The book will provide readers with a starting point into the literature on most aspects of bone metastases, but it could have been a much leaner production.

J. V. Moore R. D. Hunter